# Flunixin Meglumine Is Superior to Meloxicam for Providing Analgesia after Surgical Castration in 2-Month-Old Goats

**DOI:** 10.3390/ani12233437

**Published:** 2022-12-06

**Authors:** Victor Brusin, Maria Camila Ceballos, Pedro Henrique Esteves Trindade, Karen Camille Rocha Góis, Gabriel Conde, Virginia Tessarine Barbosa, Gustavo dos Santos Rosa, Mateus Jose Rodrigues Paranhos da Costa

**Affiliations:** 1 Faculty of Veterinary Medicine, University of Calgary, Calgary, AB T3R 1J3, Canada; 2 UNESP, Faculty of Agricultural and Veterinary Sciences, Ethology and Animal Ecology Research Group (ETCO), Jaboticabal 14884-900, SP, Brazil; 3 UNESP, Faculty of Veterinary Medicine and Animal Science, Graduate Program in Animal Biotechnology, São Paulo State University, Botucatu 18618-681, SP, Brazil; 4 UNESP, Faculty of Agricultural and Veterinary Sciences, Department of Animal Science, Jaboticabal 14884-900, SP, Brazil; 5 CNPq—National Council for Scientific and Technological Development, Brasília 71605-001, DF, Brazil

**Keywords:** non-steroidal anti-inflammatory drugs, pain, von Frey monofilament test, Anglo-Nubian goat kids, animal welfare

## Abstract

**Simple Summary:**

Farm animals are exposed to various painful procedures during their productive lives, making it necessary to implement anesthetic and analgesic protocols. However, there are few studies evaluating the effectiveness of these drugs in domestic species. We evaluated the effects of meloxicam and flunixin meglumine in pain sensitivity of goat kids subjected to surgical castration with local anesthesia. The von Frey monofilament test was used to evaluate pain sensitivity in three body regions: four points of the scrotum (dorsal and ventral; left and right lateral); medial region of the pelvic limb, gracilis muscle; and hypogastric region of the abdomen. Reactions were recorded before castration, immediately after castration, and once-daily for three consecutive days after castration. Meloxicam-treated goats experience increased pain sensitivity in the scrotal region and gracilis, particularly at 1 day after castration. However, flunixin meglumine resulted in lower pain reactions, indicating more effective pain relief.

**Abstract:**

Farm animals are exposed to various painful procedures during their productive lives, making it necessary to implement anesthetic and analgesic protocols. However, there are few studies evaluating the effectiveness of these drugs. Our objective was to compare the analgesic effects of two nonsteroidal anti-inflammatory drugs (NSAIDs): meloxicam (MEL) and flunixin meglumine (FLU), in goat kids subjected to surgical castration under local anesthesia. Anglo-Nubian goat kids (60 days old) were allocated into two groups: MEL (n = 9), and FLU (n = 8), each administered 5 min before starting castration. All had been previously subjected to local anesthesia with lidocaine, injected bilaterally into the testes, plus subcutaneous in the scrotal raphe. Pain sensitivity was evaluated using the von Frey monofilaments test. Reactions were recorded before castration (M0), immediately after castration (M1), and once-daily for three consecutive days post-castration (M2, M3, and M4, respectively). Pain assessments were conducted in three body regions: at four points of the scrotum (dorsal and ventral; left and right lateral; R1); medial region of the pelvic limb, gracilis muscle (R2); and hypogastric region of the abdomen (R3). MEL goats had considerably greater pain reaction in R1 and R2 over time, mainly in M2; therefore, FLU was a more effective analgesic than MEL, resulting in less pain reaction.

## 1. Introduction

“Pain is an unpleasant sensory and emotional experience associated with actual or potential tissue damage or described in terms of such damage” [1]. This harmful stimulus to the body is perceived by the central nervous system, which responds through defence mechanisms involving modifications in both behaviors and the nervous system, attempting to restore homeostasis [2,3]. Human and non-human animals experience pain resulting from tissue damage and subsequent inflammatory response [4], negatively affecting their welfare [5]. Although many routine management procedures in livestock production systems, e.g., castration, dehorning, and tail docking, are painful, most are performed without anesthetic or analgesic protocols for pain management [6,7].

Despite the increasing commercial availability of various analgesics and anesthetics, there are challenges restricting their use in farm animals, including regulatory concerns, delayed onset and short duration of action, repeated animal handling, etc. [7]. The most commonly used analgesics are nonsteroidal anti-inflammatory drugs (NSAIDs), such as flunixin or meloxicam, and are currently the best options for on-farm analgesia [7,8]. NSAIDs are organic acids that inhibit cyclooxygenase, decreasing prostaglandins and inhibiting inflammatory processes [8]. Therefore, NSAIDs are useful analgesics for painful procedures [9,10,11].

The effects of NSAIDs depend on the species, associations with other drugs, and pharmacokinetics of the specific NSAID used [12,13,14]. For example, after intravenous administration, meloxicam concentration was significantly higher in ovine than caprine plasma up to 48 h after its application, and not detectable in goats at 72 h after administration [15]. Conversely, Königsson et al. [16] reported that the pharmacokinetics of flunixin meglumine following intravenous administration in goats were similar to those in other species, e.g., cattle and sheep.

It was recently reported that goat kids in the Netherlands are often dehorned without anesthetics or analgesics [17]. Additionally, most procedures were incorrectly performed, resulting in hemorrhage, exudative inflammatory signs, and necrosis of the epidermis around the remnant horn bud and subcutaneous tissue. 

In a survey conducted by the Goat Veterinary Society, experienced veterinary surgeons (who disbudded ~ 2000 goat kids/annum) reported no adverse reactions when analgesics (mainly meloxicam) were used [18]. Consequently, the British Veterinary Association and the Goat Veterinary Society released a policy statement endorsing meloxicam for post-operative analgesia. Additionally, in goat kids given local lidocaine anesthesia and dehorned, those also given flunixin meglumine spent more time in activity behaviors (e.g., exploring, playing, and feeding) than a saline-treated control [19]. It was recently reported that goats receiving transdermal flunixin after surgical castration might have pain mitigated, but authors suggested that a single dose may not be sufficient to reduce the physiological indicators of pain [20]. Therefore, both NSAIDs (meloxicam and flunixin meglumine) efficiently controlled pain in goat kids. However, to our knowledge, a contemporaneous comparison of these two products for pain control after castration of goat kids has not been reported. 

Our objective was to compare the effects a single dose of two non-steroidal anti-inflammatory drugs, meloxicam and flunixin meglumine, on pain sensitivity in goat kids subjected to surgical castration under local anesthesia. This study was a first step to overcome the resistance to the use of analgesia under commercial conditions, aiming to raise awareness among goat breeders to adopt this strategy to mitigate pain during aversive procedures.

## 2. Materials and Methods

The study was conducted in accordance with National Council for the Control of Animal Experimentation (CONCEA) guidelines and approved by the Committee of the Ethical Use of Animals of the Faculty of Agricultural and Veterinary Sciences, São Paulo State University (UNESP), Jaboticabal, São Paulo state, Brazil (Protocol nº 006470/17).

### 2.1. Study Locations, Animals, and Experimental Groups

Anglo-Nubian goat kids belonging to the Goat Teaching and Research Facilities of the Faculty of Agricultural and Veterinary Sciences in Jaboticabal, São Paulo state (n = 12) and Faculty of Veterinary Medicine and Animal Science in Botucatu, São Paulo state (n = 5), both UNESP institutions, were used. At the time of castration and von Frey test assessments, the goat kids were approximately 60 days old and weighed 7.28 ± 0.73 kg. Goats were randomly assigned to two experimental groups receiving either flunixin meglumine (FLU) or meloxicam (MEL). Furthermore, all goats were given local anesthesia (lidocaine 20 mg/mL, without vasoconstrictor; Lidovet®, Laboratório Bravet, Engenho Novo, RJ, Brazil) before castration. The von Frey monofilament test was performed by one evaluator blinded to the treatments given to goats. 

### 2.2. Orchiectomy Procedure

The orchiectomy procedures were performed by two surgeons, one in the Goat Teaching and Research Facilities of the Faculty of Agricultural and Veterinary Sciences in Jaboticabal, São Paulo, Brazil, and the other in the Faculty of Veterinary Medicine and Animal Science in Botucatu, São Paulo state, Brazil. All goats were kept in a 1.50 × 1.02 m pen, located within a covered shed, throughout the experiment. All goats were fasted for 12 h before surgery, which was always performed in the morning. On the day of surgery, the goat first received a total of 5 mg·kg^−1^ local anesthesia (lidocaine, without vasoconstrictor; Lidovet®, Laboratório Bravet, Engenho Novo, RJ, Brazil), injected bilaterally into the testes, and subcutaneously in the scrotal raphe. After 2 min, eight goats (FLU) received a dose of 1.1 mg·kg^−1^ of flunixin meglumine (Flunixina^®^, Ourofino, Cravinhos, SP, Brazil), and the remainder (MEL: n = 9) received a dose of 0.5 mg·kg^−1^ of meloxicam (Maxicam^®^, Ourofino, Cravinhos, São Paulo state, Brazil), with both NSAIDs given intramuscularly. After castration, all goats received (intramuscularly) 1.0 mL of antibiotic (Terramicina LA^®^, Oxitetraciclina, Zoetis) for every 10 kg of body weight.

Five minutes after administering anesthesia and analgesia, the goats were placed in an elevated supine position, with the hind limbs positioned close to the belly. Antisepsis was performed with 70% alcohol and 2% chlorhexidine gluconate (Vic Pharma^®^, Taquaritinga, SP, Brazil). Testes were firmly stabilized in the scrotal region and a skin incision was made directly over each testis, in the most ventral aspect of the scrotum. Then, the vaginal tunics were incised, and slight traction was used to expose the testis. The epididymis tail ligament was severed to release the visceral vaginal tunic and cremaster muscle, as well as to expose the spermatic cord and vas deferens. The spermatic cord was clamped with two straight clamps approximately 3 cm apart. Then, in the tissue of the spermatic cord next to the remaining clamp, the spermatic cord was transfixed and ligated with 3–0 nylon thread and swaged-on needle, the spermatic cord was sectioned between the clamps, and the testis was removed. After the procedure, povidine iodine (Rioquímica^®^, São José do Rio Preto, SP, Brazil) was flushed into the scrotum and a larvicide and healing agent (Max Prata, Vansil^®^, Descalvado, SP, Brazil) applied. After surgery, each goat was returned to its pen.

### 2.3. Local Nociceptive Assessment

**Figure 1:**
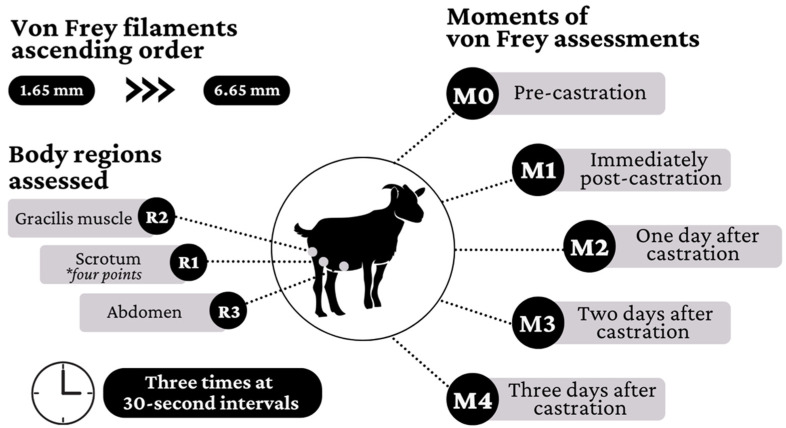
Body regions assessed (R1 = at four points of the scrotum (dorsal and ventral; left and right lateral); R2 = medial region of the pelvic limb (gracilis muscle); and R3 = hypogastric region of the abdomen.) and moments of von Frey filament test assessments (M0 = pre-castration, M1 = immediately post-castration, M2, M3 and M4 = 1, 2, and 3 days after castration, M4, respectively).

The local nociceptive thresholds after castration procedures were measured using von Frey monofilaments [21], useful for mimicking clinical conditions such as increased skin sensitivity, neuropathic pain, postoperative pain, and inflammation [22]. The filaments, applied in ascending order (from the smallest to the largest thickness) to the cutaneous surface, exert a pressure converted into a unit of force. When there was no aversive response to the application of the smallest diameter filament (e.g., 1.65 mm), the next larger diameter filament was used, and so on, until obtaining an aversive response (raising and lowering the limb, stroking, or vocalize) or the largest diameter filament was used (6.65 mm). The filaments were applied three times at 30-s intervals, in three body regions: R1—at four points of the scrotum (dorsal and ventral; left and right lateral); R2—medial region of the pelvic limb (gracilis muscle); and R3—hypogastric region of the abdomen. The von Frey assessments were performed five times: pre-castration (M0), immediately post-castration (M1), and 1, 2, and 3 days after castration (M2, M3, and M4, respectively), as summarized in Figure 1.

### 2.4. Statistical Analyses

Statistical analyses were performed using R software in the RStudio integrated development environment (Version 1.0.143^©^ 2009–2016, RStudio, Inc., Boston, MA, USA). The filaments values were converted into force (g) using the manufacturer’s table [21]. First, the normality of force values of von Frey filaments at each moment, and for each experimental group, were visually assessed, using the “histogram” function of the “lattice” package. Based on the graphs, distributions of all data were considered non-normal. 

The Friedman test (“friedman.test” function in the “stats” package) was used to investigate changes in sensitivity within the FLU and MEL groups over time (M0 vs. M1 vs. M2 vs. M3 vs. M4). To avoid the influence of ties in the ranking established by the Friedman test, the value of the probability of rejection of the null hypothesis was corrected with the Bonferroni procedure (function “pairwiseSignTest” of the package “rcompanion”). Then, intergroup strength values (FLU vs. MEL) were compared in each evaluation moment, using the unpaired and two-tailed Wilcoxon test (“wilcox.test” function of the “stats” package). Finally, *p*-values were assumed significant when *p* < 0.05.

The analysis to estimate the sample size was conducted by setting 0.80 for power and 0.05 for significance level for the two-tailed hypothesis test of two samples (“power. *t*-test” function in the “stats” package). For the most relevant variable of the study [force values (g) of the von Frey filament test, just after castration at scrotum], the delta (258.6605) was reached by subtracting the mean calculated for the meloxicam group (49.77311) and flunixin meglumine group (308.4336) and the common standard deviation of both groups (178.9688). According to the aforementioned limits, the sample size was estimated to be at least 8 goats per group (≅8.59).

## 3. Results

Before castration procedures (M0), five of nine goat kids subjected to treatment MEL were not reactive to the von Frey test (accepting the highest force value, 446.683 grams) when applied to the scrotum and hypogastric region of the abdomen, and six of nine, when the test was performed in the gracilis muscle. Similarly, six of eight goats subsequently treated with FLU did not react to the von Frey test when applied to the scrotum and hypogastric region of the abdomen before being subjected to the castration procedure; with seven of eight not reacting to the test when applied to gracilis muscle. In general, goats that responded to the von Frey test with greater intensity in both treatments (MEL IDs 6, 7, 8, and 9; and FLU IDs 13, 15, and 17) before castration had more intense pain reactions in subsequent von Frey assessment moments (Supplementary Tables S1–S3). There was no significant difference between groups of goats assigned to MEL versus FLU treatments before the surgical procedure was performed (median = 446.683 g, the highest value) in all sites where pain assessments were performed.

**Figure 2:**
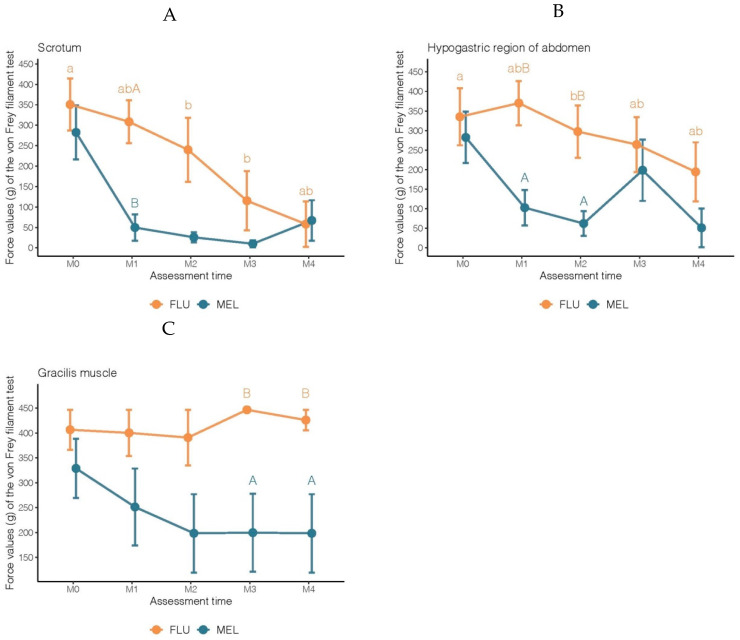
Mean force values (g) ± standard deviation of the von Frey filament test obtained in goat kids subjected to a surgical castration procedure under anesthesia and receiving analgesic protocols with meloxicam (MEL: 0.5 mg/kg^−1^ IM) or flunixin meglumine (FLU: 1.1 mg/kg^−1^ IM). Assessments were performed just before castration (M0), just after castration (M1), 1, 2, or 3 days after castration (M2, M3, and M4, respectively), in three body regions: (**A**) scrotum (average of four points), (**B**) hypogastric region of the abdomen, and (**C**) gracilis muscle. Notes: Lowercase letters indicate significant differences over time (M0 vs. M1 vs. M2 vs. M3 vs. M4) within the same treatment group (FLU and MEL) and each body region (A: Scrotum, B: Hypogastric region of abdomen, and C: Gracilis muscle). Capital letters represent significant differences between treatments (MEL vs. FLU) within each body region.

Significant differences over time in the von Frey test were observed only for MEL when measurements were performed on the scrotum and the hypogastric region of abdomen, with increased pain sensitivity on the first day after surgery (M2) compared to M0 (Table 1). There were also significant differences in pain sensitivity between MEL and FLU in the scrotum in M1, in the gracilis muscle in M3 and M4, and in the hypogastric region of the abdomen in M1 and M2. In all cases, goats in the MEL group had higher medians of pain sensitivity than FLU (Table 1, Figure 2).

## 4. Discussion

Surgical castration is often performed on male goat kids, usually without anesthesia or analgesia [17]. Our objective was to compare the efficiency of two NSAIDs (meloxicam and flunixin meglumine) for pain relief in goat kids surgically castrated with anesthesia. To maximize validity, all handling procedures and other factors, including animal species, route of administration and associations with other drugs [15,16,23] that could alter the effects of the analgesics tested were controlled, ensuring that the results were directly related to the effects of the tested analgesics to control postoperative pain [24].

Goats that received flunixin meglumine did not have increased postoperative pain sensitivity, whereas those that received meloxicam exhibited pain soon after the surgery, presenting higher sensitivity than the flunixin meglumine-treated goats until 72 h post-surgery. This was partially in agreement with Shukla et al. [15], who reported that plasma meloxicam concentrations in goats were low at 48 h after intravenous application, and not detectable at 72 h after drug administration. Conversely, flunixin meglumine was detected in plasma samples after transdermal administration in goats up to 96 h after administration [16].

The lower efficacy of meloxicam in the present study corroborated previous reports. Kells et al. [25], assessing pain in castrated lambs after receiving a combined protocol of anesthesia and analgesia (using intramuscular meloxicam), did not report statistical differences in decreased pain-related behaviors compared to animals not subjected to anesthesia and analgesia. Similar results were also observed with piglets, as reported by Hansson et al. [26], and Kluivers-Poodt et al. [27]. In these studies, piglets were subjected to castration, comparing at least two groups; one received only meloxicam and the other a combination of lidocaine and meloxicam. According to Kluivers-Poodt et al. [27], meloxicam alone was effective only on the first day after castration. Conversely, Mélendez et al. [28] reported that a combination of lidocaine and meloxicam partially mitigated pain in surgically castrated calves, as it reduced substance P concentrations, white cell count, and the frequency of tail flicks after castration. 

The efficacy of flunixin meglumine plus lidocaine for pain relief in goat kids partially corroborated previous results. Park et al. [29] compared not castrated and castrated Korean cattle bull calves, receiving or not receiving lidocaine combined with flunixin meglumine, to determine the effects of this combination on various indicators of pain and inflammation. In that study, the combination of both drugs did not reduce elevated pain but tended to decrease elevated inflammation in castrated animals, suggesting that additional treatment may be required to alleviate pain. Similarly, Webster et al. [30] reported a positive effect of a combination of lidocaine and flunixin meglumine on post-castration performance, plasma cortisol concentrations, and behavior of dairy calves, with attenuation of signs of pain and stress. However, we are not aware of published studies comparing the effects of meloxicam and flunixin meglumine as pain relief for goat kids.

The combined use of anesthetic and analgesic products is characterized as multimodal pain management, aiming to potentialize the pain relief effects by targeting different pain receptors [31]. It is worth noting that using local lidocaine before administering either NSAID could have influenced our results. This study is a first step to promote the use of analgesia when performing painful procedures in goat kids under commercial conditions. Nevertheless, future studies should be performed to evaluate the feasibility of using at least one post-operative analgesic treatment when castrating goat kids kept under commercial production systems.

## 5. Conclusions

We concluded that combining local anesthesia (lidocaine) with a single dose of non-steroidal anti-inflammatory flunixin meglumine before goat kids’ castration resulted in less pain sensitivity reactions compared to those treated with lidocaine plus meloxicam. Therefore, we recommend combining local lidocaine with flunixin meglumine when surgical castration is performed in goat kids kept under commercial production systems.

## Figures and Tables

**Table 1:** Median, minimum, and maximum (min/max) force values (g) of the von Frey filament test obtained in goat kids subjected to a surgical castration procedure under anesthesia and receiving analgesic protocols with meloxicam (MEL: 0.5 mg/kg^−1^ IM) or flunixin meglumine (FLU: 1.1 mg/kg^−1^ IM). Assessments were performed just before castration (M0), just after castration (M1), 1, 2, or 3 days after castration (M2, M3, and M4, respectively), in three body regions: scrotum (average of four points), hypogastric region of the abdomen, and gracilis muscle.

Treatment	Assessment Time				
	M0	M1	M2	M3	M4
Scrotum					
MEL	286.287 ^a^ (28.840/446.683)	3.63 ^abB^ (0.17/281.840)	0.166 ^b^ (0.068/75.858)	0.117 ^b^ (0.068/75.858)	0.166 ^ab^ (0.068/446.683)
FLU	446.683 (0.068/446.683)	281.838 ^A^ (0.068/446.683)	446.683 (0.023/446.683)	0.215 (0.005/446.683)	0.407 (0.005/446.683)
Hypogastric region of abdomen					
MEL	446.683 ^a^ (28.840/446.683)	3.63 ^abB^ (0.17/281.840)	0.166 ^bB^ (0.068/281.838)	0.166 ^ab^ (0.068/446.683)	0.166 ^ab^ (0.068/446.683)
FLU	446.683 (0.166/446.683)	446.683 ^A^ (0.068/446.683)	364.260 ^A^ (0.023/446.683)	286.287 (0.023/446.683)	100.875 (0.023/446683)
Gracilis muscle					
MEL	446.683 (28.840/446.683)	446.683 (0.166/446.683)	0.166 (0.068/446.683)	11.749 ^B^ (0.068/446.683)	0.166 ^B^ (0.068/446.683)
FLU	446.683 (125.892/446.683)	446.683 (75.858/446.683)	446.683 (0.028/446.683)	446.683 ^A^ (446.683/446.683)	446.683 ^A^ (281.838/446.683)

Within a row, medians with different lowercase letters differed over time (M0 vs. M1 vs. M2 vs. M3 vs. M4) within each body region and treatment. Within a column, medians with different capital letters represent significant differences between treatments (MEL vs. FLU) within a body region (scrotum, hypogastric region of the abdomen, and gracilis muscle).

## Data Availability

The data that support the findings of this study are available from the corresponding authors upon reasonable request.

## Supplementary Materials

The following supporting information can be downloaded at: https://www.mdpi.com/article/10.3390/ani12233437/s1, Table S1: Individual responses of goat kids to the von Frey test applied to the scrotum according to the treatment and assessment moment; Table S2: Individual responses of goat kids to the von Frey test applied to the gracilis muscle according to the treatments and assessment moment; Table S3: Individual responses of goat kids to the von Frey test applied to the hypogastric region of the abdomen according to the treatments and assessment moment.
