# Supplementary material for: Flunixin Meglumine Is Superior to Meloxicam for Providing Analgesia after Surgical Castration in 2-Month-Old Goats
Source: Animals (Basel). 2022 Dec 6;12(23):3437. doi: 10.3390/ani12233437 (PMC9736442; doi:10.3390/ani12233437)
Supplement: Supplementary file 1 [file animals-12-03437-s001.zip › animals-2025231-supplementary.pdf]

Supplementary Table S1. Individual responses of goat kids to the von Frey test applied to the scrotum according to the treatments and assessment moment.

Treatments	Animals	Von Frey force				
		Before castration M0	Just after castration M1	One day after castration M2	Two days after castration M3	Three days after castration M4
**Meloxicam**	164	446,683	3,630	75,858	75,858	446,683
	160	446,683	3,630	75,858	2,041	125,892
	144	446,683	125,892	75,858	8,511	28,840
	136	446,683	3,630	3,630	3,630	2,041
	30	446,683	281,838	0,166	0,166	0,166
	17	28,840	28,840	0,068	0,068	0,068
	29	28,840	0,166	0,068	0,068	0,068
	30	125,892	0,166	0,166	0,068	0,068
	9	125,892	0,166	0,068	0,068	0,068
**FlunixinMeglumine**	162	446,683	281,838	28,840	0,407	2,041
	12	446,683	281,838	446,683	0,023	0,166
	142	446,683	281,838	75,858	0,005	0,407
	170	446,683	281,838	28.840	0,005	0,407
	330	446,683	446,683	446,683	28,84	0,407
	336	0,068	0,068	0,023	0,005	0,005
	339	446,683	446,683	446,683	446,683	446,683
	335	125,892	446,683	446,683	446,683	15,136

Supplementary Table S2. Individual responses of goat kids to the von Frey test applied to the Gracilis muscle according to the treatments and assessment moment.

Treatments	Animals	Von Frey force				
		Before castration M0	Just after castration M1	One day after castration M2	Two days after castration M3	Three days after castration M4
**Meloxicam**	164	446,683	446,683	446,683	446,683	446,683
	160	446,683	446,683	446,683	446,683	446,683
	144	446,683	446,683	446,683	446,683	446,683
	136	446,683	446,683	446,683	446,683	446,683
	30	446,683	446,683	0,166	11,749	0,166
	17	446,683	28,840	0,068	0,068	0,068
	29	28.840	0,166	0,068	0,068	0,068
	30	125,892	0,166	0,166	0,068	0,068
	9	125,892	0,166	0,068	0,068	0,068
**FlunixinMeglumine**	162	446,683	446,683	446,683	446,683	446,683
	12	446,683	446,683	446,683	446,683	446,683
	142	446,683	446,683	446,683	446,683	446,683
	170	446,683	446,683	446,683	446,683	446,683
	330	446,683	446,683	446,683	446,683	446,683
	336	125,892	75,858	0,028	446,683	281,838
	339	446,683	446,683	446,683	446,683	446,683
	335	446,683	446,683	446,683	446,683	446,683

Supplementary Table S3. Individual responses of goat kids to the von Frey test applied to the hypogastric region of the abdomen according to the treatments and assessment moment.

Treatments	Animals	Von Frey force				
		Before castration M0	Just after castration M1	One day after castration M2	Two days after castration M3	Three days after castration M4
**Meloxicam**	164	446,683	3,630	75,858	446,683	446,683
	160	446,683	281,838	281,838	446,683	3,630
	144	446,683	281,838	75,858	446,683	8,511
	136	446,683	75,858	125,892	446,683	2,041
	30	446,683	0,166	0,166	0,166	0,166
	17	28,840	281,838	0,068	0,068	0,068
	29	28,840	0,166	0,068	0,068	0,068
	30	125,892	0,166	0,166	0,068	0,068
	9	125,892	0,166	0,068	0,068	0,068
**FlunixinMeglumine**	162	446,683	446,683	28,840	446,683	5,495
	12	446,683	281,838	446,683	125,892	446,683
	142	446,683	446,683	281,838	75,858	75,858
	170	2,041	446,683	281,838	125,892	125,892
	330	446,683	446,683	446,683	446,683	8,511
	336	0,166	0,068	0,023	0,023	0,023
	339	446,683	446,683	446,683	446,683	446,683
	335	446,683	446,683	446,683	446,683	446,683
